# Biotechnological Production of Sustainable Microbial Proteins from Agro-Industrial Residues and By-Products

**DOI:** 10.3390/foods12010107

**Published:** 2022-12-25

**Authors:** Bojana Bajić, Damjan Vučurović, Đurđina Vasić, Rada Jevtić-Mučibabić, Siniša Dodić

**Affiliations:** 1 Department of Biotechnology, Faculty of Technology Novi Sad, University of Novi Sad, Bulevar cara Lazara 1, 21000 Novi Sad, Serbia; 2 Institute for Food Technology Novi Sad, University of Novi Sad, Bulevar cara Lazara 1, 21000 Novi Sad, Serbia

**Keywords:** single-cell protein, microbial protein, microbial biomass, biotechnology, agro-industrial residues, agro-industrial by-products

## Abstract

Microbial proteins, i.e., single-cell proteins or microbial biomass, can be cultivated for food and animal feed due to their high protein content and the fact that they represent a rich source of carbohydrates, minerals, fats, vitamins, and amino acids. Another advantage of single-cell proteins is their rapid production due to the growth rate of microorganisms and the possibility of using agro-industrial waste, residues and by-products for production through this renewable technology. Agro-industrial residues and by-products represent materials obtained from various processes in agriculture and agriculture-related industries; taking into account their composition and characteristics, as well as vast amounts, they have an enormous potential to generate sustainable bioproducts, such as microbial proteins. This review aims to summarize contemporary scientific research related to the production of microbial proteins on various agro-industrial residues and by-products, as well as to emphasize the current state of production of single-cell proteins and the importance of their production to ease the food crisis and support sustainable development.

## 1. Introduction

According to the Food and Agriculture Organization of the United Nations (FAO), the world’s population is projected to reach 9.7 billion by 2050. A deficiency in food sources represents a severe problem due to the growing population. More than a billion people are undernourished worldwide due to insufficient natural resources, increasing discrepancies in food demand and supply, as well as the effects of global warming, human health and soil erosion. Proteins are nitrogen and essential amino acid sources necessary for humans and animals to build new structural and functional proteins such as enzymes and hormones, and are necessary for both growth and cell regeneration [1,2,3,4]. Food’s nutritional/nutritive value, also known as protein quality, relies on its amino acid content and the utilization of specific amino acids. Therefore, concentrations and ratios of amino acids affect the quality of particular proteins, and the biological quality is greater if the proportion of indispensable amino acids (histidine, isoleucine, leucine, lysine, methionine, phenylalanine, threonine, tryptophan, and valine) is greater [5].

As a result of food deficiency concerns, alternative proteins may be developed as a replacement for conventional proteins. These alternatives require less intensive production methods. Alternative proteins include microbial proteins, insect-based proteins, cell-based meat, plant-based meat substitutes and dairy alternatives [3,6]. Microbial proteins, commonly known as single-cell proteins (SCP), are derived from several species of microorganisms but are most commonly derived from microalgae, fungi, yeast, or bacteria. Professor Carroll Wilson created the term “single-cell protein” at the Massachusetts Institute of Technology (MIT) in 1966. SCP represents microorganism biomass or protein extract that can be used in animal and human nutrition [7,8,9]. Aside from proteins, single-cell protein products may contain free amino acids, carbohydrates, lipids, vitamins, and minerals [10]. It is considered that compared to animal or plant proteins, SCP has a high nutritional value [11]. SCP production had its first culmination in Germany during World War I when *Saccharomyces cerevisiae* was grown on molasses for human consumption as a protein supplement [12].

Single-cell proteins are utilized mainly as protein-rich food supplements or ingredients for human and animal nutrition. Furthermore, they are used for paper and leather processing and as a foam stabilizer [13]. Production of single-cell proteins for feed is connected to animal farming and agriculture; however, it allows the utilization of uneatable materials after arable land use and increases resource efficiency [14]. In comparison with agricultural proteins, SCP production is more environmentally friendly, consumes less water, requires smaller land areas and its effect on climate change is much less pronounced than in the case of agriculturally derived proteins [15]. When used as feed, SCP may serve as a replacement for traditional protein supplements such as fishmeal and soymeal. For monogastric animals, soybean meal is the best and most significant source of dietary protein [16]. Fishmeal, i.e., ground dried forage fish and/or fish trimmings and waste, has been the preferred protein ingredient in aquaculture. Since aquaculture is competing with fishmeal use in swine, poultry and other animal diets, fishmeal production cannot scale with the growth of all these industries without jeopardizing forage fish fisheries. More suitable protein ingredients are needed during the industry’s expansion in order to maintain feed performance and benefit aquaculture health [17]. Production of microbial proteinaceous biomass has several advantages compared to conventional animal farming and crops. Microorganisms, due to their short doubling time (algae and molds, 2–6 h; bacteria and yeasts, 0.33–2 h), produce protein much more efficiently than any farm animal or plant (1–2 years and a couple of months, respectively). Moreover, microorganisms have a relatively high protein content on a dry mass basis (30–80% *w*/*w* dependent on the microorganism used), and the nutritional value of the protein is good. A broad spectrum of raw materials can be used as a substrate in SCP, including low-value agro-industrial residues and by-products. Microbial proteins can be grown in vast quantities in relatively small continuous fermentation processes using a relatively small land area. SCP production is also independent of climate and seasonal and climatic variations. Microorganisms are also more easily genetically modified than plants and animals [18,19,20,21]. Further, SCP complies with the essential amino acid requirements for human nutrition recommended by the FAO/WHO [22].

Therefore, this review aims to consolidate data published in the scientific literature in recent years related to single-cell protein producing microorganisms, the use of various agro-industrial residues and by-products in the production of microbial proteins, and the most significant characteristics of their production and application. Furthermore, the current state of microbial protein production and future perspectives for solving problems related to food safety, health and sustainability are presented.

## 2. Current Situation of Microbial Protein Production

As a result of a growing population worldwide, economic development and urbanization, as well as rises in protein-rich diets, such as Atkins and Keto, meat consumption has increased exponentially over the past 50 years, reaching over 328 million metric tons in 2021 [23,24]. Likewise, the UN predicts that demand for protein will have increased by more than 50% by 2050 compared to 2020 levels [25]. However, inflation due to conflicts worldwide is increasing the price of meat and cereals used as animal feed [26]. Because of the rising import prices of these commodities, the EU is at risk of food insecurity [27]. Moreover, industrialized animal agriculture is among the top 2–3 most significant contributors to the world’s most pressing environmental issues, such as water use, air pollution, deforestation and biodiversity loss [28]. As much as 75% of agricultural land is used for raising and feeding livestock [29], which only provides 1/3 of the global protein supply [30].

Given the planet’s limited natural resources, this is cause for concern, prompting entrepreneurs to rethink the efficiency of protein production. Recent years have seen several meat alternatives emerge, with some available in supermarkets across the world. Soybean products are the most common, even though they are costly, have arguably an unappetizing taste and heavily impact the environment, contributing to almost 20% of tropical deforestation [31]. Insect-based products represent another solution, but lack in consumer acceptance due to cultural differences around the world [32]. Finally, microbial protein obtained from cultivating sugar-fed bacteria, yeast, filamentous fungi and algae could reduce the agricultural land use, thus drastically decreasing deforestation and related CO_2_ emissions [33]. Eating microbial protein is familiar, since humans have been consuming products linked to microbes (beer, bread, yoghurt and cheese) long before they even knew of their existence.

**Figure 1:**
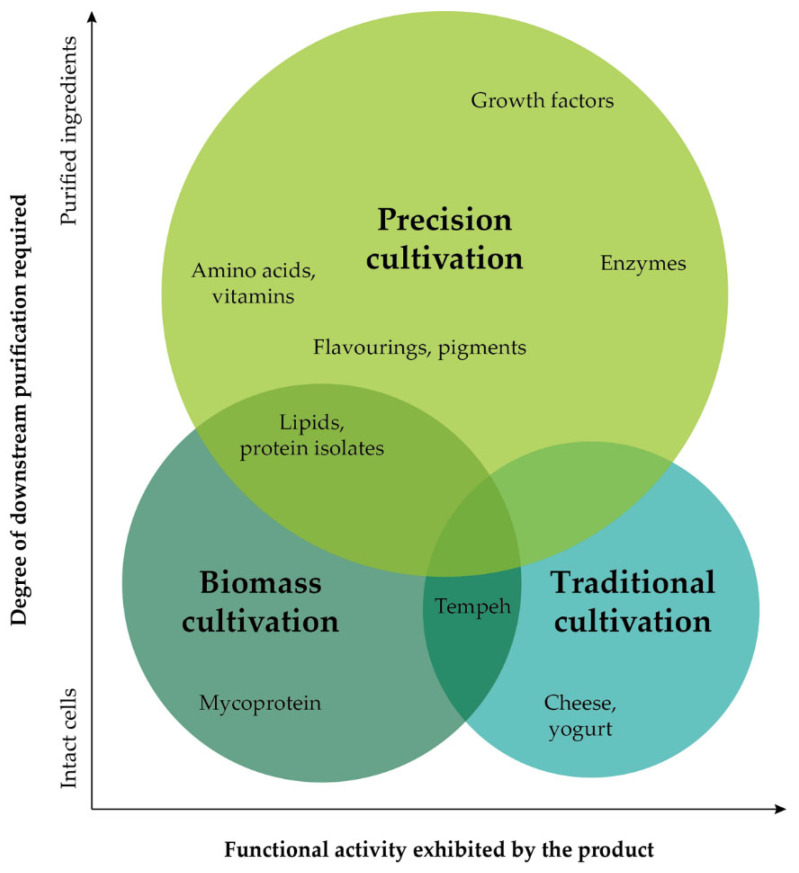
A conceptual landscape of cultivation-derived and cultivation-enabled products [35].

All of the aforementioned explains why there is a significant expansion in the alternative protein industry. According to a GFI (Good Food Institute) company database, 88 fermentation companies are focused exclusively or predominantly on alternative proteins, and $1.69B was raised by dedicated alternative protein fermentation companies in 2021, which is up 285% from 2020, representing 60% of all-time investment [34]. There are three primary ways of utilizing cultivation in the alternative protein industry (Table 1) with a spectrum of products (Figure 1).

As seen from Table 1 and Figure 1, cultivation technology provides the possibility of producing a huge diversity of products, such as meat, dairy and egg replacements, seafood, fats and oils, infant food, pet food, and many more, as well as enhancing plant-based products across these food categories. Traditional methods have been practiced for thousands of years to create foods such as wine and cheese. Using live microbes, they can modify plant-based components, enhancing their taste, nutritional value and texture. The power of microorganisms possessing high amounts of protein to grow quickly is exploited by biomass cultivation to effectively generate a lot of protein fast. It is then possible to use the microbial biomass as a food ingredient, either with a minimal degree of processing or with its cells intact. The purpose of precision cultivation is to produce specific functional ingredients by using microbes as factories for generating the desired component. Compared to primary protein, these ingredients are utilized in much smaller quantities but higher purities because of their powerful influence on the sensory and functional characteristics of the final product.

Despite being a mature technology and all of the innovations so far, it is considered that the new cultivation platforms have only scratched the surface due to the immense physiological diversity of microorganisms. Biotechnologists have scientific strategies to discover new types of food during this fast-approaching era of transitioning away from animal-based proteins.

## 3. Biotechnological Production of Single-Cell Proteins

### 3.1. SCP Producing Microorganisms

The selection of the appropriate production microorganism is of great importance in every biotechnological process. Precisely because of this, microorganisms for SCP production are chosen based on oxygen requirements and heat generation during fermentation, foam character, growth rate, productivity, and/or yield of specific low-cost substrates, tolerance to temperature and pH, genetic stability, growth morphology, end product composition and structure, having regard to ease of protein recovery and purification [36,37]. Although several examples of SCP production by heterotrophic bacteria are given in the scientific literature, most heterotrophic SCP produced on an industrial scale has been synthesized with yeast or fungi [14]. Among microorganisms used for SCP production, microalgae have the highest protein content (60–70% *w*/*w*), followed by bacteria (30–80% *w*/*w*), yeasts (30–50% *w*/*w*) and protists (10–20% *w*/*w*) [38].

#### 3.1.1. Fungi

*Fusarium venenatum* is one of the most commercially well-known fungal SCP species and is utilized to produce a meat alternative, Quorn^TM^. It was successfully launched in 1985 and is currently one of the most well-known SCP products. In Finland, a process known as Pekilo was created in the 1970s and 1980s to make feed protein from the sugars found in the sulphite waste liquor of paper mill effluents utilizing the filamentous microfungus *Paecilomyces variotii* [18,39,40]. Despite being marketed as animal feed, the product was also tested as a supplement for meat-based foods such as sausages and meatballs [39]. Fungi’s potential to utilize a variety of organic components for growth is one property that makes them advantageous for the production of SCP. *Rhizopus oryzae* was used in submerged and solid-state fermentation to utilize residual fruit and vegetable waste, as reported by Ibarruri et al. [41]. Other species of fungi used for SCP production on various substrates are *Aspergillus flavus*, *A. niger, A. ochraceus, A. oryzae*, *Cladosporium cladosporioides*, *Monascus ruber*, *Penicillium citrinum*, and *Trichoderma viride* [40,42,43]. The possibility of mycotoxin production with certain species of fungi, such as *Fusarium, Alternaria* and *Aspergillus* species, during cultivation requires consideration, however [44]. Another food safety hazard associated with mycoproteins (SCPs produced from fungi) are allergens. While data is limited, adverse reactions to mycoproteins have been reported in individuals with a history of mold allergies [6]. Fungal SCP can be used to improve the nutritional value and functional features of food items, such as texture and emulsifying and foaming capacity, in addition to being an excellent source of protein-rich nutrients in and of itself [45].

#### 3.1.2. Yeast

Biotechnological applications of yeasts, a heterogeneous group of eukaryotic fungi, are currently restricted to a limited number of species, such as *Candida utilis, Kluyveromyces marxianus, Yarrowia lipolytica* and *Pichia pastoris*, among which *Saccharomyces cerevisiae* has a prevailing position [46]. Yeasts have the ability to grow on miscellaneous substrates, have high protein content (45–55% dry weight), contain vitamins of the B-complex, and represent one of the most extensively used microorganisms [19,47]. Aside from their ability to grow at acidic pH and their size, which makes them easier to harvest, the essential advantage is familiarity and acceptability due to long-term use in traditional fermentation [47]. Additionally, yeasts typically have higher lysine content than bacteria, and the opposite is true for methionine [48].

*Saccharomyces cerevisiae*, also known as brewer’s or baker’s yeast, is traditionally used for production of yeast extracts production. Additionally, it is used to produce salty spreads such as Marmite, Cenovis and Vegemite [14]. The genus *Candida* has been used to produce SCP in multiple studies using different agroindustrial wastes and residues, such as yellow wine lees [49], tuber wastes [50], pineapple cannery effluent [48], salad oil manufacturing wastewater [51], orange peel residues [19], and sugarcane bagasse hemicellulosic hydrolysate [52]. It should be noted that several *Candida* species are opportunistic human pathogens and the most common causative agents of candidiasis are *C. albicans*, *C. glabrata*, *C. parapsilosis*, *C. tropicalis* and *C. krusei* [53]. Due to its pathogenic character, there is not much literature on *C. krusei* industrial biotechnology implementation. However, *C. krusei* has a wide range of biotechnological applications, and the fact that it is found in many traditional foods such as milk products and tapai suggests that no mycotoxin is secreted in the finished fermented product. Regardless, there is still a need to take extra precautions during the production of SCP to ensure that there are no live yeast cells in the final product [54]. *Yarrowia lipolytica* is phylogenetically remote from other well-researched yeast species. The FDA has given GRAS (Generally Recognized as Safe) status to its metabolites, and the European Food Safety Authority (EFSA) approved its biomass as a novel food in 2019 [14,55]. *Kluyveromyces marxianus* is a lactose-utilizing yeast in whey and whey permeate [56] and represents a great candidate for SCP production; it is being widely used as a feed organism [46].

#### 3.1.3. Algae

Algae are generally grouped into two categories based on their morphology and size—microalgae and macroalgae. Microalgae, as the name indicates, are microscopic photoautotrophic microorganisms. They use energy from sunlight to convert carbon dioxide and water into organic materials for cellular functions [57,58]. In addition to CO_2_ and light, sugars can be added to boost growth and biomass production rates and yields. This is known as the mixotrophic production mode [17].

*Arthrospira maxima* and *Arthrospira platensis*, which are commonly known as *Spirulina*, together with *Chlorella* are the most extensively used [8,14]. There are some limitations to human consumption when it comes to algae. The most important limit is the presence of the algal cell wall because humans lack the enzyme cellulase; hence they cannot digest the cellulose component of the cell algal wall. Therefore, cellulose digestion is necessary before the final product is consumed for SCP to be used as human food [8]. A variety of methods can disrupt the cell wall: chemical (such as organic solvents or acids), enzymatic (such as cellulases) and physical and mechanical (such as bead milling, high-pressure homogenization, or microfluidics) [17,59]. The aforementioned treatments are usually applied to some rigid cell-walled species such as green microalgae *Chlorella vulgaris, Nannochloropsis oculata* and *Haematococcus pluvialis* to release the intracellular biomolecules. Milder recovery methods are needed for microalgae with thinner cell walls, like *Arthrospira platensis* and *Porphyridium cruentum* [59]. However, cellulose digestion can be omitted if the SCP is used as feed for cattle as they have cellulose-degrading symbiotic bacteria and protozoa in their rumen [8].

Microfluidics was applied for the cell rupture of *Chlorella vulgaris*, which was later used as feed for juvenile Atlantic salmon. The cell-rupture processing improved the digestibility of major energy-yielding nutrients (e.g., proteins, lipids, carbohydrates). The applied method of cell-rupturing had a very minimal effect on the biochemical composition of *C. vulgaris* meal compared to a whole-cell meal [60].

*Aphanothece microscopica Nägeli*, a cyanobacterium with a higher protein content than traditional foods such as meat, eggs, and wheat meal, was studied for the production of SCP with the effluent of parboiled rice as a source of nitrogen and organic matter. The apparent digestibility of 82.12% in young white male Wistar rats indicates that *Aphanothece* biomass is a possible source of SCP [10]. However, in some cases, allergic reactions to spirulina-derived products have resulted in anaphylaxis after consumption [61,62].

#### 3.1.4. Bacteria

Bacteria have traits that make them suitable to produce microbial protein, such as rapid growth, short generation time, and ability to grow on a variety of raw materials ranging from carbohydrates (starch and sugars) to gaseous and liquid hydrocarbons (including methane and petroleum fractions) to petrochemicals (such as methanol and ethanol) [37].

*Cellulomonas* and *Alcaligenes* are the most frequently used bacterial species as an SCP source [8,37]. *Methylococcus capsulatus* is a methanotrophic bacteria that has been used commercially for SCP production from fossil-based feedstocks, i.e., natural gas and synthetic nitrogen. Methanotrophs use methane as their only source of carbon and energy while assimilating nitrogen from cultivation media leading to protein production [1,63]. Methanol-obligate bacterium *Methylophilus methylotrophus* was commercially used in the production of PRUTEEN^®^. However, commercial production was terminated mainly due to economic considerations involving increased oil prices [64].

Zhu et al. reviewed the use of phototrophic bacteria (PSB) *Rhodobacter capsulatus* for SCP production using food waste fermentation liquid as a substrate. The data in their study showed that an excess of carbon source inhibited microbial metabolic activities, which reduced SCP biosynthesis. The inhibited activities include the phosphorylation process of PSB, viability, transport of Ni and Co, and osmotic stress tolerance. The optimal regulation of the carbon source could stimulate the environmental behaviour of PSB, resulting in a greater SCP yield [65]. Hydrogen-oxidizing bacteria *Alcaligenes eutrophus, Seliberia carboxydohydrogena* and *Ralstonia eutropha* were proven to be a potential protein source due to the high protein content, valuable amino acid content and availability of proteolytic enzymes [66].

The substrate, fermentation conditions, type of bacteria, and post-fermentation processing all affect the chemical composition of the bacterial biomass [63]. The use of bacterial SCP is limited because harvesting protein from bacteria is costly due to their smaller cell size. Therefore, bacterial cells must be flocculated to give a higher solids slurry before centrifugation. Further, there is a poor public acceptance of bacteria as food [8]. Hülsen et al. suggested using a biofilm photobioreactor to cultivate mixed culture phototrophic bacteria in pre-settled red meat processing wastewater as a potential way to reduce the harvesting cost substantially. Compared to suspended systems, the biofilm bioreactor’s main disadvantage is its capital cost [67].

#### 3.1.5. Mixed Cultures of Microorganisms

The use of mixed cultures of microorganisms has been suggested in order to increase biomass yield and improve protein quality [68,69]. However, the interactions among strains during mixed fermentation still need to be clarified. *Candida tropicalis*, *Aspergillus oryzae* and *Trichoderma koningii* were evaluated for the production of SCP feed using orange waste. Zhou et al. established synergistic and antagonistic effects during mixed fermentation: *T. koningii* and *A. oryzae* mutually promoted each other. However, the growth of *C. tropicalis* was inhibited by *A. oryzae* and *T. koningii* as polygalacturonase and carboxymethyl cellulase accumulated [70].

A mixed yeast consortium of *Kluyveromyces lactis* and *Rhodotorula graminis* was proven efficient for SCP production from waste milk, a major by-product of the dairy industry that contains many nutrients such as lactose, vitamins, casein, and minerals. When compared to the sequential culture, the mixed culture, under optimized conditions, enhanced SCP productivity and reduced Total Organic Carbon (TOC) [71].

Several different combinations of microorganisms have been suggested for SCP production using whey as a substrate: *Candida utilis* and *Torulopsis cremoris* [68], *Kluyveromyces marxianus* and *Candida krusei* [69], *Kluyveromyces marxianus* and *Saccharomyces cerevisiae* [72].

Sugar beet pulp, a waste product of the sugar beet industry, supplemented with molasses and glucose, was also used as a substrate for SCP production by mixed culture sequential fermentation of *Candida utilis* and *Brevibacterium lactofermentum*. Compared to mono-cultures of *B. lactofermentum* and *S. cerevisiae*, mixed culture leads to greater production of amino acids, crude protein, and true protein [73].

### 3.2. Substrates for SCP Production

The type of substrate utilized and the composition of the cultivation media determine how much SCP is produced, keeping in mind that selecting an appropriate substrate has a direct impact on the effectiveness of the bioprocess [74]. A variety of substrates have been utilized to produce SCP and can be categorized as high-energy resources (gas oil, natural gas, ethanol, methanol, n-alkanes, and acetic acid), renewable plant resources (starch, sugar, and cellulose), various wastes (sulfite waste liquor, molasses, whey, milk, and fruit waste), and carbon dioxide [8,36].

A significant quantity of residue is produced annually by industries based on agriculture that, if discharged into the environment without safe disposal, might lead to the degradation of the environment and be damaging to both human and animal health. Agro-industrial residues, by-products and waste encompass agricultural residues (leaves, stalks, seed pods, stems, straw, molasses, husks, bagasse, seeds, shell, pulp, stubble, peel, roots, etc.), which can be further divided into field residues (present in the field after the process of crop harvesting), process residues (present after the crop is processed), and industrial residues (produced in different branches of food processing industries such as juice, chips, meat, confectionary, and fruit industries, and including potato peel, orange peel, cassava peel, coconut oil cake, soybean oil cake, etc.) [75].

For waste material to be a useful substrate, it should be abundant, non-toxic, cheap, and able to support rapid growth and multiplication of the implemented microorganism [37]. When choosing the waste material for SCP production, the following aspects should be considered: accessibility of waste, cost of pre-treatment, transportation cost, and the concentration of protein in the final microbial biomass [76]. Using different biodegradable wastes as a substrate in the fermentation process can be an alternative method for reducing the environmental impact of these substances [74]. Moreover, using agro-industrial residues in SCP production lowers the main production cost [9,77]. Even though the application of agro-industrial by-products, residues and waste streams as raw materials in the production of microbial biomass has many advantages, it can also affect the occurrence and accumulation of certain toxic compounds, such as pesticides or heavy metals. As a result, applying synthetic cultivation media with defined composition and high-grade carbon sources ensures the safety and high quality of the final product [78].

Table 2 summarizes different types of microorganisms used for SCP production on agro-industrial residues, wastes and by-products.

Depending on the type of constituent with the largest share, Spalvins et al. divided agricultural waste into four main groups: mono and disaccharide-rich sources, starch-rich sources, structural polysaccharides-rich sources, and protein or lipid-rich sources. Microorganisms can metabolize mono- and disaccharide-rich substrates (such as molasses, dairy, and fruit processing wastes) with high product yields and mild pre-treatment, significantly decreasing the entire production cost. However, in order to use starch-rich substrates, they must first be hydrolyzed into monosaccharides, which could raise the overall cost. Finally, in order to generate very high protein concentrations in the final biomass from protein-rich sources used in SCP production, hydrolysis employing proteolytic microbial enzymes is required [76].

A trend that has developed recently in SCP production is the exploitation of fungal species for bioconversion of lignocellulosic wastes [90]. For the lignocellulosic waste to be used as a fermentation substrate, it is necessary to involve a pretreatment to reduce the recalcitrant nature of the lignocellulosic material. The main goal of the pretreatments is to solubilize and remove one or more structural components from biomass, specifically cellulose, lignin, and hemicellulose. Pretreatment increases substrate porosity with lignin redistribution and permits the maximum exposure of saccharification enzymes to cellulose and hemicellulose surfaces to achieve improved hydrolysis with minimal energy consumption [9,19], i.e., pretreatment makes the feedstock more accessible for the enzymatic hydrolysis. Since enzymes have a substantial impact on the cost of the process, optimizing hydrolysis conditions including enzyme dosage, hydrolysis time, and solids concentration is essential [40]. The use of lignocellulosic substrates for SCP production makes it possible to produce a higher-value product compared to second generation biofuels and chemical production [18].

Ram horns are a significant percentage of the waste products from the meat industry in Turkey, and because of their high organic loads, they are typically disposed of in landfills via municipal sewers. In their 2002 study, Kurbanoglu and Algur investigated hydrolyzed ram horn as a substrate for the fermentation of SCP by *Escherichia coli*, *Bacillus subtilis*, and *Bacillus cereus*. In order to prepare the substrate for fermentation, ram horns underwent pretreatment (ground and then impregnated with 6 N HCl) and the obtained hydrolysate was successfully used to produce SCP while minimizing pollution [93].

The economic feasibility of producing SCP using wheat straw in three different SCP processes was analyzed by Voutilainen et al. in 2021. The production of lignocellulosic sugars from wheat straw involved pretreatment with steam explosion, enzymatic hydrolysis of the pretreated slurry, and solid-liquid separation of the sugars. *Paecilomyces variotii*, *Fusarium venenatum*, and *Candida utilis* were cultivated on the obtained sugars. As the primary cost factors, plant capacity, investment, raw material costs, and enzyme price were noted, meaning that the high-value dietary protein can be used to offset the high cost of lignocellulosic sugar production [18].

Elevated chemical oxygen demand, biochemical oxygen demand, total solids, organic carbon, heavy metals, etc. are all linked to effluents from the paper and pulp industry. The quantity and the composition of pulp and paper mill effluent are determined by the manufacturing process used. Khumcai et al. conducted a study in which they tested a viable approach to remediating pulp and paper mill effluent using predominant indigenous bacterial species (*Streptomyces tuirus* OS1) and the feasibility of using the bacteria as SCP. In the short period of the bioremediation process with multi-metal tolerant *S. tuirus* OS1, most physicochemical characteristics were found to be within acceptable limits at 35˚C. The biomass obtained from the bioremediation process was also proven to have a high crude protein content (5.3 g/L at 35 °C) which, after in-vitro and in-vivo research, may lead to it being considered as SCP for food and feed use [95].

In a study by Bertasini et al. it was shown that a mixture of agricultural digestate (rich in macro and micronutrients) and candy production effluent (rich in sugars) was a suitable alternative for *Saccharomyces cerevisiae* production of SCP under aerobic conditions. The superior performance of *S. cerevisiae* under aerobic conditions compared to anaerobic conditions is compatible with the scientific understanding of this yeast’s metabolism [38].

One of the primary agricultural industrial byproducts in the manufacturing of cheese or casein is whey. Whey creates serious environmental and health risks because of its high organic content and extensive production. Its main ingredients are lactose (about 74% of the dry weight), proteins (about 10% of the dry weight), mineral salts (about 8% of the dry weight), and fat (about 1% of the dry weight) [96]. The high organic load is caused by the presence of whey nutrients, both organic and inorganic. This also illustrates why whey is viewed as a potential resource for producing a wide range of value-added products [21]. Several SCP processes have been developed using whey as a substrate for lactose-utilizing biomass production to help reduce the problems of waste disposal from cheesemaking processes such as *Kluyveromyces marxianus* [56,97] and a mixed culture of bacteria and yeasts (*Lactococcus lactis* subsp. *lactis*, *Lactococcus lactis* subsp. *lactis* biovar *diacetylactis*, *Lactococcus lactis* subsp. *cremoris*, *Leuconostoc mesenteroides* subsp. *cremoris*, *Lactobacillus kefyr*, *Candida kefyr*, *Saccharomyces unisporus)* [98].

Due to the high impurity level, raw glycerol, one of the main by-products of biodiesel manufacturing, has relatively low value. Therefore, to transform the crude product into pure glycerol, a valuable component of commercial grade, use of energy-intensive and expensive methods is required. The amount of glycerol that is readily available on the market has significantly increased as a result of the growth in biodiesel manufacturing, and as a result, it will be crucial to find new uses for raw glycerol [99]. Different *Yarrowia lipolytica* strains were grown on raw glycerol to yield biomass with a protein content ranging from 42.1% to 46.8%, which was within the acceptable range for fodder yeast (40–52%). Given the high protein concentration, employing raw glycerol as a substrate for SCP formation suggests that this residue can be effectively utilized to create a valuable product [100].

As a viable substitute substrate for *Cupriavidus necator* cultivation, synthesis gas (also known as syngas) produced from biomass gasification was investigated. *C. necator* is a hydrogen-oxidizing bacterium which could utilize hydrogen as the electron donor and oxygen as the electron acceptor to fix carbon dioxide into protein. Syngas has the potential to serve as a substrate for the creation of SCP, as the authors’ study showed. Additionally, the syngas-to-protein bioconversion process was recommended as a potential way to selectively recover CO from syngas due to *C. necator* inability to metabolize CO [101].

### 3.3. SCP Production Process

SCP production involves several basic steps [102]:

1. Preparation of an adequate medium with a suitable carbon source,
2. Prevention of contamination of the chosen fermentation medium and the bioreactor,
3. Production of the desired microorganisms,
4. Separation of microbial biomass and its processing.

**Figure 2:**
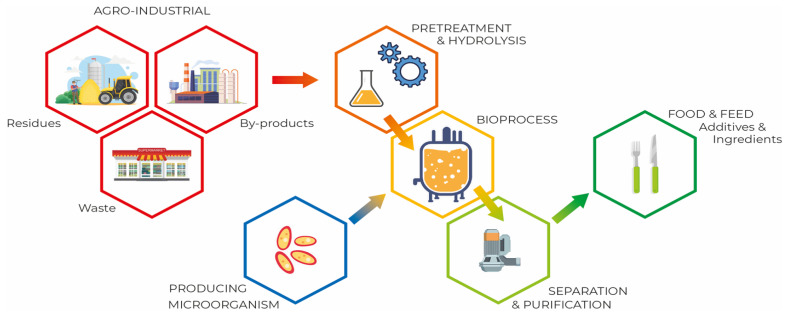
Microbial protein production process.

Figure 2 schematically shows SCP (microbial biomass or microbial proteins) production from agroindustrial residues and by-products.

Depending on the chosen medium, preparation may include pretreatment steps such as shredding or pulverizing and filtering to remove solids, followed by heat treatment, acid or enzyme hydrolysis that converts the pulp into soluble reducing sugars. Pretreatment can be classified into four different groups: (a) physical pretreatment, (b) chemical pretreatment, (c) physicochemical pretreatment, and (d) biological pretreatment. Pretreatment of the substrate improves the availability of bound nutrients and reduces the size of the components. However, depending on the pretreatment needed, this can significantly increase the cost of the manufacturing process [9,19,102,103]. One of the significant factors in protein synthesis by microbial biomass, due to its structural properties, is the nitrogen source. Ammonia, urea, nitrate, ammonium salts, and organic nitrogen present in different substrates are nitrogen sources valuable for the growth of microorganisms [74].

The substrate for fermentation is chosen based on factors such as price, availability, and the cost of downstream processing [104]. The biomass obtained after the bioprocess is subjected to separation and purification procedures that may include washing, cell disruption, extraction of proteins and purification [8].

SCP production aims to maximize cellular growth and co-product yields in economically viable approaches [17]. The production of microbial biomass as SCP by cultivating microorganisms on abundantly available agricultural and industrial wastes is done by a submerged, semi-solid or solid-state fermentation process [8,37].

Fermentation refers to the biological process of turning complex substrates into simple chemicals, due to the action of a variety of microorganisms. Temperature, pH, the nature and composition of the medium, dissolved oxygen, carbon dioxide, and the mode of bioreactor operation (such as batch, fed-batch, or continuous) all have an impact on the fermentation process. Changes in these variables may have an impact on the rate of fermentation, the product spectrum, yield, the organoleptic features of the product (aesthetics, flavor, texture, and mouthfeel), the production of toxins, nutritional value, and other physicochemical characteristics [13].

In submerged fermentation (SmF), the substrate is always in a liquid state, and contains the nutrients necessary for biomass growth [37]. SmF, also known as liquid fermentation, is best suited for microorganisms that require high moisture content, such as bacteria. An advantage of SmF is that the purification of products is easier [105].

A major factor which determines the physical properties of a fermentation broth is the dominant morphological form of fungi being cultivated in SmF. With greater viscosity of a fermentation fluid, it is typically more difficult and expensive to acquire adequate momentum transfer to produce a homogenous, well-mixed cell suspension. This may lead to a limitation of nutrients, not only O_2_, especially if the fermenter is operated in continuous or fed-batch mode. Moreover, if the peripheral fermentation fluid is slow-moving or stagnant, precise temperature control becomes very difficult [106].

A biotechnological process in which microorganisms grow on a solid material (the substrate itself or inert support impregnated with liquid medium) without the presence of free liquid is recognized as solid-state fermentation (SSF) [75]. The solid substrate in the SSF process provides nutrients for the microorganisms and acts as a cell anchor. However, some nutrients might not be present in the substrates or present in subpar amounts. Therefore, it would be required to replenish them externally in such circumstances. In addition, some substrates, such as lignocelluloses, have also traditionally undergone pre-treatment before being used in SSF processes, which makes them more amenable to microbial growth [107].

In order to optimally utilize the microorganism metabolism and growth, the SSF substrate needs to have an adequate proportion of water content [108]. Due to the fact that fungi prefer dry substrates naturally, unlike other microbes, SSF offers the greatest potential when used with fungi. Since moisture is a necessary factor for microbial growth, the concept of water availability in substrate becomes important [109]. The water activity of substrates, due to the strong influence of water on microbial activity, determines the types of microorganisms that can grow in SSF [110]. The low thermal conductivity of substrates used for SSF decreases heat removal and increases its accumulation. Heat removal is one of the critical issues in SSF, which is why most studies are focused on maximizing heat removal [111]. Problems with heat transfer can be solved by minimizing the substrate bed height. However, this is only applicable to small-scale SSF. Adequate mixing of the substrate with sparged oxygen can also help; it aids in the homogeneity of the bed but also ensures adequate heat and mass transfer [112]. Bioreactors commonly used for SSF can be divided into four types based on the agitation system employed or the type of aeration. These are the tray, packed bed, horizontal drum, and fluidized bed [113].

Semi-solid fermentation is a form of solid-state fermentation where the amount of free liquid is raised to improve nutrient availability and regulate fermentation [114]. Higher moisture content is known to fill substrate voids, which restricts the microorganism gaseous mass transfer. Microorganism growth is similarly constrained in cases of reduced moisture content. Although moisture is crucial for the semi-solid fermentation process, other parameters such as temperature, pH, the type of biomass, and properties such as particle diameter, surface area, and particle voidage might also be crucial for a successful semi-solid fermentation [115]. This modification of the SSF process, which originated from the brewing industry in ancient China, features high transport efficiency and system productivity, easy operation and low secondary pollution [116].

Bioreactors typically operate in one of the three modes: batch, continuous, or fed-batch. During batch fermentation, which can be considered as a closed system, no substrate is added after the initial charge, and the product is not extracted until the end of the process. At the start of the process, the sterilized medium in the bioreactor is inoculated with microorganisms, thus initiating the bioprocess. Generally, the batch operation mode is not considered commercially attractive. On the other hand, a continuous mode of operation, in which the product is continuously withdrawn, and the substrate is continuously added, is considered more economical. In the fed-batch mode of operation, the substrate is slowly fed to the reactor, but no product is removed until the end of the process. One of the advantages of the fed-batch mode of operation is avoiding substrate overfeeding, which can inhibit the growth of microorganisms [117].

The microbial biomass is collected following fermentation and may go through further processing operations such as washing, cell disruption, protein extraction, and purification. Due to the low solids content of SmF fermentation products (1–5%), pre-concentration is usually needed to ease dehydration. This can be accomplished through centrifugation, heating, filtration, and evaporation. In order to enable further handling and save transportation expenses, the finished product should be in a dry powder state [114,118].

Fungal SCPs are primarily produced in submerged fermentations. However, there is an increasing interest in solid-state fermentation [14]. A tubular photobioreactor and so-called raceway ponds are the most commonly used microalgae cultivation systems for producing SCPs. Since sunlight is the critical factor for microalgal growth, the light supply rate directly dictates the productivity of the photobioreactor or raceway pond. However, costs currently limit large-scale SCP production from microalgae. Investments in materials and equipment are substantial, as well as the power required to mix the cultures, supply CO_2_, and remove photosynthetically produced O_2_ [58].

A single-cell protein process generally involves four main cost components: capital, fixed, raw materials, and enzymes costs. A considerable amount of the costs comes from pretreatment and hydrolysis, i.e., processing the raw material into sugars [18].

Direct use of SCP as food is restricted because of the higher nucleic acid content, which may lead to the development of gout disease in humans due to the accumulation of uric acid in the body if consumption is too high. Different techniques have been proposed for the reduction of nucleic acid content in SCP (below 2% *w/w*), such as chemical (e.g., sodium chloride, ammonium hydroxide and sodium hydroxide) and enzymatic (e.g., ribonuclease and deoxyribonuclease) treatments [72,119,120]. Even though both chemical and enzymatic treatments effectively reduce nucleic acid content, the nutritional quality of substrates may be altered by these treatments [120]. Nucleic acid content varies depending on the group of microorganisms: fungi (7–10% dry weight), yeast (6–12% dry weight), algae (3–8% dry weight) and bacteria (8–12% dry weight) [47,121].

With microbial engineering, single-cell protein products can become more competitive in terms of production costs, nutrition, and functionality and the main objective of microbial engineering should be optimizing the accumulation of biomass and production of intermediate feedstocks. In addition, utilizing microorganisms with a GRAS status is always the most acceptable alternative when employing microbial engineering in SCP production [122].

## 4. Future Perspectives and Outcomes of Microbial Protein Production

The Food 2030 research and innovation policy framework represents the strategy of the European Union for solving problems related to food safety, health and sustainability for the period up to 2030 and microbial proteins are covered by alternative proteins that could contribute to the environmentally suitable change in nutrition [123]. Specific co-benefits of developing microbial protein production from agro-industrial residues is significant for the following:

- Agriculture—by using fewer land resources for crop and animal farming, as well as valorization of agro-industrial residues,
- Food production—in a faster and more cost-effective way to ensure food security for a growing world population,
- Feed production—in larger quantities with fewer resources,
- Environmental protection (circularity and sustainability)—by cutting deforestation and biodiversity loss, reducing greenhouse gas emissions (reversing climate change), and enhancing better air and water quality,
- Human health—by decreasing malnutrition, providing healthier and sustainable diets and diversifying the offer of proteins,
- Science and economy—by enhancing research, engaging young scientists, cooperating with stakeholders and industry, fostering competitiveness, triggering innovation, business models, value-added products, goods, services, and jobs,
- Society—by changing consumer habits, breaking down barriers to dietary transition, and educating and raising awareness about healthier and more sustainable choices.

Future areas of focus for the development of alternative protein production technologies will be the identification and selection of target metabolites, the development of microbial strains, the discovery and optimization of feedstocks, the design and scale-up of bioprocesses, and the commercialization of end products [35]. For the first two a detailed screening of different candidate strains needs to be performed in order to obtain new protein products. Another important area will be getting to know the metabolic and biosynthetic pathways and functions of the desired molecule in the microbes. This is where modern computational tools and bioinformatics will have a powerful effect and major role in defining entirely new food ingredients.

Microbial engineering can be applied in different ways regarding SCP production, such as improving substrate utilization, microorganism growth, stress tolerance, and protein production, and improvement of the nutritional and functional quality of SCPs [122,124,125]. Another area of concern is that feedstocks prepared from complex raw materials usually contain toxic compounds that inhibit cell growth. The use of microbial engineering can help reduce the uptake of these toxic compounds, which would result in improved stress tolerance and cell growth [126]. Engineering metabolic pathways for the utilization of different carbon sources present in the substrate, such as xylose, has also improved growth and biomass accumulation [127]. The fishmeal substitute KnipBio Meal utilized in the aquaculture sector is a successful genetically modified SCP product approved for commercial usage [128]. Whilst microbial engineering may enhance SCP production, there are still some technological obstacles to overcome such as generally poor engineering effectiveness, especially for atypical microorganisms, and relatively poor efficiency of SCP production (including the exploitation of carbon sources, biomass accumulation, SCP yield, and the amount of nutritional and functional components). Finally, SCP microorganisms typically contain heterologous genetic components, which could cause some societal concern [122].

Feedstocks that form the cultivation media needed for microbial growth during the bioprocess also require screening and optimization. A key area for innovation within feedstocks is utilizing side streams and waste [22] from the agri-food industry. Few companies are even working on producing protein from carbon present in air [129] and even from plastic waste [130]. Since all waste materials can be gasified into CO_2_, CH_4_, or CO_2_ gases, these gases have a significant impact on climate change if they are allowed to escape into the atmosphere. However, they can be employed as carbon feedstocks to produce SCP. The utilization of gasified waste materials as carbon sources for the production of SCP significantly contributes in the transition to a circular economy [131]. On the other hand, nitrogen fixing from side (waste) streams of the Haber–Bosch process by hydrogen oxidizing bacteria has been examined for edible microbial protein production [132].

Experimental optimization is one of the main techniques used to develop novel foods, but it has been shown that these methods have significant raw material and pre-testing costs, while also having substantial environmental impacts. Therefore, other technologies have been used to decrease costs, create value, and adjust to market demands, such as artificial intelligence (AI) [133]. A wide range of sectors, including science and technology, industry, and even daily life are being significantly impacted by AI in combination with promising machine learning (ML) techniques well known from computer science [134]. Within this concept, other methodologies have been created, including genetic algorithms (GA) and artificial neural networks (ANN) [133].

The use of AI in personalized nutrition is one of the most interesting applications with the greatest growth potential. The average person nowadays is increasingly conscious of the quality of their diet and is in search of different alternatives to meet all of their needs. Using mathematical and statistical models, AI can assist the food industry in forming foods according to the needs of their customers [135]. Likewise, ML has been used to measure calorie intake, i.e., develop apps that determine the calories of a meal based on the picture taken by the user [136]. Bioprocess design has been exploited successfully for increasing scale, lowering costs and improving biotechnological processes, so it will surely have its place in the microbial protein production sector. In this context, much effort will be directed towards commercializing industrial manufacturing, since this step has been identified as a critical one for most companies in the field of alternative protein production. Additionally, developing platforms for downstream processing, continuous bioreactor operation and virtual platforms are other major themes and opportunities in bioprocess design that will surely support the advancement of microbial proteins, thus leading to a dramatic acceleration in bringing these new products to the market.

Although cultivation provides a viable and sustainable method of production, it is not without its challenges, one of them being capacity, i.e., there is not enough of it. The growth of cultivation capacity will arise from individual producers followed by partnerships with strategic entities and big multinationals in order to build the needed scale. Food manufacturers will be sourcing ingredients from startups producing them initially and then they will eventually invest in their own capabilities to produce those ingredients in the same way as well. Pooling resources through collaboration between producers will help tackle this scale problem together [35].

Given the long history of utilizing microbial cultivation in the food industry, government regulations concerning microbial protein are partially established in most countries and will need to be updated to meet the developments in the alternative protein sector. In order for a product to become available on the US market, the company will need to obtain a “no questions” letter from the FDA (Food and Drug Administration), i.e., GRAS status [137]. For the EU, the EFSA will perform a pre-market authorization procedure which includes a risk assessment according to the EU’s novel food regulation [138]. Further, governments have recognized the potential of alternative proteins and began funding and supporting open-access research, start-ups and industrial manufacturing. According to the GFI’s Global Policy Report for 2021 [139] around 360 million US dollars have been invested in alternative protein R&D, with Singapore, Israel, Canada, Europe (especially Denmark), the US and China leading the way in terms of secured means of funding. The same report has a projection of the global alternative protein market size, with annual sales growth from 250 to 500+ billion US dollars by 2050. According to the ING Research [140], the long-term development of market share between meat and meat alternatives will take decades (around 2060) for meat alternatives to surpass meat, based on a current meat alternative market annual growth rate of 10%. Additionally, the public support should be focused on establishing favorable conditions in order to get the private sector interested in investing with more confidence. However, if alternative proteins are to reap the benefits they promise, they must overcome the barrier of consumer acceptance, i.e., taste as good as or even better than conventional meat and be as affordable or cheaper.

## 5. Conclusions

The enormous potential of cultivating microorganisms can be leveraged to contribute to food system innovations in a way that will surpass sensory, nutritional, environmental, social, market and functional paradigms of existing (animal) proteins. We are only just beginning to scratch the surface of what cultivation-based approaches can offer the alternative protein industry, and consumers as well as current players in the sector are eager for innovative solutions and products made possible by this technology. This sector is expected to witness several trends, including recognition of the environmental and other benefits of this type of cultivation, acceleration of innovation by computational approaches, increasing use of hybrid products, exploration of alternative proteins from a wider range of sources, precision cultivation products becoming more common, and an increase in the frequency of products being released. Factors that would drive sales growth include supportive government and regulatory action, investment and innovation, product development, scientific advancement, scaled production capacity and increased consumer adoption. In spite of the uncertainty surrounding how exactly the industry will grow, stakeholders are already on the way to a future with sustainable food supply.

## Figures and Tables

**Table 1:** Cultivation approaches in the alternative protein industry [34].

Cultivation Approach	Product Description	Company	Country	Year Founded
Traditional cultivation	Fermented plant-based food products (conventional cheese analogues)	Väcka	Spain	2019
	Minimally processed whole-cut meat and fish alternatives grown naturally from fungal mycelium	Bosque Foods	USA	2020
	Uses raw materials of the Mediterranean Diet: grains, legumes, nuts, and seeds to drive fermentation	The Mediterranean Food Lab	Israel	2017
	Fermented plant-based yogurt optimized for gut health	Wellme	China	2021
	Pea and rice proteins fermented by shiitake mycelium	MycoTechnology	USA	2013
Biomass cultivation	Mycoprotein-based meat substitutes	Quorn	UK	1985
	Microalgae-based plant-based foods including egg, seafood, meat, and dairy replacements	Algama	France	2013
	Beef production via a high protein yeast blend.	More Foods	Israel	2019
	Mycelium-based whole cut meats, including bacon under the brand “MyBacon”	MyForest Foods	USA	2019
	Algae-based protein	Sophie’s BioNutrients	Singapore	2010
Precision cultivation	Fermentation based non-GM functional proteins for the food industry, starting with vegan ovalbumin and related proteins	Eggmented Reality	Israel	2022
	Animal-origin-free dairy proteins and fats	Maya Milk	Turkey	2021
	Plant-based meat products, under the brand “BUDS,” and dairy products, under the brand “MilkCELL,” using precision fermentation	All G Foods	Australia	2020
	Milk protein using microbial fermentation	Zero Cow Factory	India	2020
	Meat and fish proteins through precision fermentation	Paleo	Belgium	2020
	Fermentation of dairy triglycerides and synthetic polymers	Circe	USA	2020

**Table 2:** SCP producing microorganisms used on different agro-industrial residues, wastes and by-products.

Producing Microorganism	Substrate	References
Fungi		
*Aspergillus niger*	Banana, cucumber, orange, pineapple, and watermelon food wastes	[79]
*Aspergillus (Aspergillus niger, Aspergillus flavus and Aspergillus ochraceus), Fusarium (Fusarium semitectum, Fusarium sp1, Fusarium sp 2), Monascus ruber, Penecillium citrinum* and *Cladosporium cladosporioides*	Rice bran	[43]
*Trichoderma viride* and *Geotrichum candidum*	Orange peel	[80]
*Agaricus blazei, Auricularia fuscosuccinea* and *Pleurotus albidus*	Brewer-spent grain and grape bagasse	[81]
*Aureobasidium pullulans*	Almond hulls waste	[82]
*Rhizopus oryzae*	Fruit and vegetable discards	[41]
Yeast		
*Candida utilis*	Orange peel residues	[19]
*Candida utilis*	Rice polishings	[83]
*Candida utilis*	Pineapple cannery effluent	[48]
*Candida utilis*	Salad oil manufacturing wastewater	[51]
*Candida tropicalis*	Sugarcane bagasse hemicellulosic hydrolysate	[52]
*Candida lipolytica*	Olive fruit wastes	[84]
*Candida tropicalis, Aspergillus oryzae* and *Trichoderma koningii*	Orange waste	[70]
*Galactomyces candidum*	Biogas slurry	[85]
*Yarrowia lipolytica*	Food waste from the feed of anaerobic digestion reactor	[86]
*Saccharomyces cerevisiae*	Fruits and vegetables wastes (banana peel, citrus peel, potato peel, and carrot pomace)	[87]
*Saccharomyces cerevisiae*	Candy Production Effluent	[38]
*Saccharomyces cerevisiae*	Date palm waste	[88]
*Trichosporon cutaneum, Candida tropicalis Pichia stipitis, Candida guilliermondii* and *Saccharomyces cerevisiae*	Sugar beet pulp	[89]
*Kluyveromyces marxianus* and *Candida krusei*	Whey	[69]
*Rhizopus oligosporus* and *Candida utilis*	Wheat bran	[90]
Algae		
*Aphanothece microscopica Nägeli*	Parboiled rice effluent	[91]
Bacteria		
*Rhodobacter capsulatus*	Carbohydrate-rich food waste	[65]
*Bacillus licheniformis*	Potato starch processing waste	[92]
*Bacillus cereus, Bacillus subtilis, Escherichia coli*	Ram horn hydrolysate	[93]
*Bacillus subtilis*	Soya bean hull	[94]
*Streptomyces tuirus*	Pulp and paper mill effluent	[95]

## Data Availability

Not applicable.
